# World NTD Day 2022 and a new Kigali Declaration to galvanise commitment to end neglected tropical diseases

**DOI:** 10.1186/s40249-021-00932-2

**Published:** 2022-01-28

**Authors:** Thoko Elphick-Pooley, Dirk Engels, Uniting to Combat NTDs

**Affiliations:** 1 Hove, England, UK; 2 Commugny, Vaud, Switzerland

**Keywords:** Neglected tropical diseases, Kigali Declaration, World Health Organization NTD roadmap, World NTD Day, 100% Committed

## Abstract

The World Health Organization’s first roadmap and the London Declaration on neglected tropical diseases (NTDs) have allowed an unprecedented expansion of interventions to control and eliminate this group of infectious diseases that primarily affects vulnerable or marginalised communities. The 2021–2030 NTD roadmap sustains a further acceleration of interventions but also introduces a broader and more ambitious agenda, calling to be accompanied by a new political declaration. Sponsored by the Government of Rwanda, the Kigali Declaration on neglected tropical diseases will be launched in 2022 to renew and reinvigorate commitments to end NTDs, also in the wake of the current setback caused by the COVID-19 pandemic. Starting on World NTD Day 2022, a global campaign “100% Committed” will call on a broad range of stakeholders to sign the declaration and make bold financial and political commitments towards achieving the 2030 roadmap and Sustainable Development Goals’ targets for NTDs.

## Background

Unprecedented progress has been made in recent years against neglected tropical diseases (NTDs), a group of 20 poverty-related diseases that debilitate, disfigure, and can kill. Between 2015 and 2019, well over a billion out of the 1.7 billion people at risk have consistently benefitted from NTD treatments. Forty-three countries have eliminated at least one NTD so far, 600 million people no longer require treatment for NTDs and cases of some of these diseases that have plagued humanity for centuries, such as sleeping sickness and Guinea worm disease, are at an all-time low. All this provides the proof that ending NTDs is possible [1].

Two events stand out as game-changers underpinning this success, the launch of WHO’s first NTD roadmap 2012–2020 and the signing of the London Declaration on Neglected tropical diseases, both in January 2012. The first NTD Road map was to boost the scaling up of the integrated approach WHO had conceived in 2005 for the (by then 17) NTDs, by providing clear, measurable targets that all partners could agree on. The London Declaration was to rally broad support in a diverse and fragmented partner landscape for the elimination of 10 diseases for which this target was most likely to be reached. A strong WHO closely collaborating with a committed partner alliance in which the medicine-donating pharmaceutical sector has played a crucial role has led to the important achievements that we see today [2].

January 30th has been celebrated as an anniversary of the signing of the London Declaration on NTDs since 2012. The Uniting to Combat Neglected tropical diseases partnership composed of London Declaration signatories has marked the day, either by releasing annual progress reports on NTDs or by hosting high-level events [3]. In 2019, 30th January was informally established as *World NTD Day* through the leadership of the Government of the United Arab Emirates and sponsorship of the Crown Prince Court of Abu Dhabi. In May 2021, WHO’s 74th World Health Assembly formally endorsed the recognition of January 30th as World Neglected tropical diseases Day (‘World NTD Day’), commemorating the simultaneous launch of the first NTD road map and the London Declaration on NTDs on which one of the world’s biggest public-private sector partnerships was founded to end NTDs [4].

### WHO’s 2021–2030 NTD roadmap and the need for a new Declaration on Neglected tropical diseases

Building on achieved progress, WHO launched its second 2021–2030 NTD roadmap in January 2021, to further accelerate programmatic action, but also to introduce important paradigm shifts to ensure long-term impact, such as the need to intensify cross-cutting approaches—both within and beyond the health sector, and to change operating models to facilitate country ownership and sustainability [5].

The launch of a new NTD roadmap also implicitly called for a new high-level political declaration in order to mobilise political will and secure commitments to deliver the goals set out in the WHO NTD roadmap and achieve the Sustainable Development Goal (SDG) target 3.3 on NTDs (a reduction by 90% of people requiring interventions against NTDs by 2030).

The Kigali Declaration on NTDs, sponsored by the Government of Rwanda, will be the successor to the ground-breaking London Declaration on NTDs that galvanised a vast array of NTD stakeholders—endemic and donor countries, philanthropists, private-sector companies, non-governmental organisations, academia, and research organisations—into coming together to commit to prioritising NTDs. The Kigali Declaration will emulate this and go some steps further by putting in focus country ownership of NTD programmes, their integration and mainstreaming in the health sector, and cross-sectoral collaboration to tackle the underlying determinants of poverty-related diseases, to ensure long-term sustainability and impact [6].

In line with these paradigm changes, the Kigali Declaration gives a central place to national governments of endemic countries, and brings new stakeholder groups to the table, such as regional political bodies, parliamentarians, and city mayors and local government leaders of affected countries. All with the aim to enhance awareness and priority setting from a regional and national perspective as well as commitment to ensuring equitable health coverage and improving living conditions in marginalised, NTD affected settings. In the latter perspective, support by local governance authorities is crucial to put cross-sectoral action into practice and tackle the underlying determinants of NTDs [7]. Support by parliamentarians will ensure the link between their constituencies and the national government [8].

Another powerful stakeholder group recently engaged in NTD advocacy is youth, [9] who have been instrumental in raising awareness and creating community-led demand for services in other health areas, such as HIV/AIDS.

Although COVID-19 has lately set back progress against NTDs, new innovative and broadened partnerships can ensure further progress in the right direction.

### Building on World NTD Day 2022

Now universally and formally recognised, World NTD Day 2022 will also be the 10th anniversary of the London Declaration. It therefore provides a perfect opportunity to highlight all that has been achieved to date through unprecedented public-private partnership and collaboration, and kick off the signing process for the Kigali Declaration, setting the scene for renewed commitments.

COVID-19 has added to growing fatigue in the fight against other infectious diseases, especially chronically endemic ones. The pandemic has also resulted in shifts in the funding landscape. In this new climate, there is need to elevate NTDs and highlight positive, ambitious, and winnable NTD goals, for the international community to remain 100% committed to ending NTDs. “100% Committed” will therefore be the name of the global campaign that will aim to secure commitments behind the Kigali Declaration.

The 100% Committed movement will be led by countries affected by NTDs, championed by civil society, influencers and people affected by NTDs, and will call on a wide range of stakeholders and young leaders around the world, to sign the Kigali Declaration on NTDs and pledge commitments. Without reinvigorated commitments, hard-fought progress may indeed be unravelled, and millions of people again exposed to these diseases. Progress made so far proves that ending NTDs is possible within our lifetime with appropriate efforts and resources–now is the moment for leaders to realise this and take action.

## Conclusion

The initial NTD roadmap and the London Declaration on neglected tropical diseases have given a boost to implementation of NTD interventions allowing to come close to WHO’s 2020 targets. The launch of WHO’s second NTD roadmap 2021–2030 implored a new high-level political declaration to reiterate and invigorate commitments to end NTDs by 2030. As the Government of Rwanda has agreed to sponsor such a declaration, the Kigali Declaration on Neglected tropical diseases will be launched in 2022 and will be preceded by a global campaign to solicit an expanded group of stakeholders that are 100% Committed to ending NTDs to sign the declaration and announce their commitments.
